# Effect of reduction temperature on the structure and hydrodesulfurization performance of Na doped Ni_2_P/MCM-41 catalysts

**DOI:** 10.1039/c9ra01582e

**Published:** 2019-05-17

**Authors:** Nan Jiang, Fuyong Zhang, Hua Song

**Affiliations:** Provincial Key Laboratory of Oil & Gas Chemical Technology, College of Chemistry & Chemical Engineering, Northeast Petroleum University Daqing 163318 Heilongjiang P. R. China jiangnandq@163.com songhua2004@sina.com +86-6503167; State Key Laboratory of Physical Chemistry of Solid Surfaces, Collaborative Innovation Center of Chemistry for Energy Materials, National Engineering Laboratory for Green Chemical Productions of Alcohols, Ethers and Esters, College of Chemistry and Chemical Engineering, Xiamen University Xiamen 361005 P. R. China 867881282@qq.com

## Abstract

The removal of sulfur compounds from petroleum is increasingly important because of the environmental pollution caused by sulfur compounds. In this work, Na doped Ni_2_P/MCM-41 catalysts were successfully prepared, and their hydrodesulfurization (HDS) performances were assessed using dibenzothiophene (DBT) as a model molecule. Moreover, the effects of reduction temperature (450–600 °C) on the structure and HDS performance of the Ni_2_P/Na-MCM-41 catalysts were studied. Results showed that: (a) the reduction temperature of the catalyst could be as low as 450 °C due to Na doping, which is about 200 °C lower than that of the conventional temperature-programmed reduction method (650–1000 °C); (b) increasing the reduction temperature lead to an increase in the diameter of Ni_2_P particles, which was demonstrated by size distribution analysis; (c) the HDS performance of the Ni_2_P/Na-M41-*T* catalysts increases with reduction temperature and 99.2% DBT conversion was observed for Ni_2_P/Na-M41-600, whereas the hydrogenation route of the catalysts decreased with increasing the reduction temperature, which indicates the lower reduction temperature favored the direct desulfurization pathway (DDS).

## Abstract 2

The removal of sulfur compounds from petroleum is increasingly important because of the environmental pollution caused by sulfur compounds.

## Graphical abstract

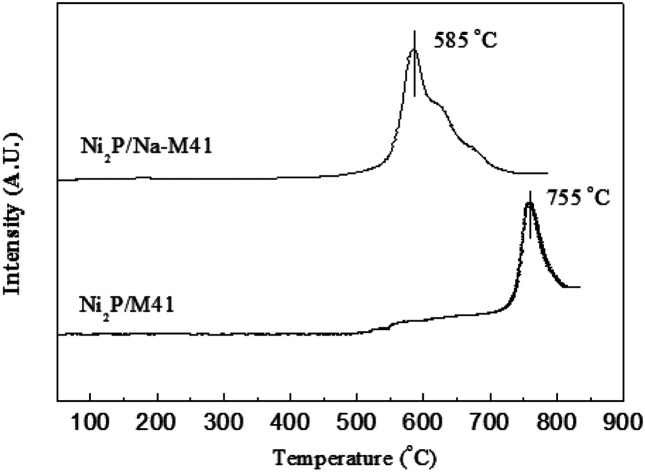

## 1. Introduction

The removal of sulfur from gasoline and diesel fuels has been the subject of intensive investigations in recent years. Sulfur-containing aromatic compounds such as thiophenes, benzothiophenes, dibenzothiophenes (DBT) and their alkylated derivatives from petroleum fractions in most cases were removed through the hydrodesulfurization (HDS) process.^1^ Among them, DBT and its alkylated derivatives are the most difficult sulfur-containing molecules to hydrodesulfurize.^2^ Supported transition metal phosphides have recently received extensive attention as a new family of non-sulfided HDS catalysts due to their high activity and stability for the HDS of petroleum feedstocks.^3,4^ Among these HDS catalysts, supported Ni_2_P has shown better HDS performance and thus are good candidates for deep HDS,^5–7^ and it was found that the HDS activity of Ni_2_P catalysts was remarkably enhanced when it was supported on MCM-41. In conventional TPR method, the oxidic precursors were obtained mainly by the impregnation of (NH_4_)_2_HPO_4_ (or NH_4_H_2_PO_4_) and Ni(NO_3_)_2_ solutions, then dried, calcined. Then the Ni_2_P phase was obtained by reducing the oxidic precursors in flow of H_2_,^8,9^ however, the reduction temperature is higher (≥650 °C) owing to the strong P–O–P bond. This means a strong investment for energy consumption during the preparation process, which limits the practical application of this Ni_2_P/MCM-41 catalyst. Therefore, the development of a simple method for preparing Ni_2_P/MCM-41 catalysts under mild conditions is an interesting direction and received a great deal of research. Generally, for the synthesis of Ni_2_P/MCM-41 catalyst under mild conditions, the methods of ‘Changing phosphide precursors’ that using different phosphide precursors such as NH_4_H_2_PO_2_ and NiCl_2_·6H_2_O to reduce the reduction temperature have been frequently used, because the reduction temperature could be effectively decreased by using low-state phosphide instead of high-state phosphide. It is well known that the reduction of nickel phosphide precursors and their dispersion on the support can be modified by the addition of some additives to the support.^10^ Among many different materials, Na-containing supports have attracted special attention in the field of hydrodesulfurization catalysis. Rodrigo^11^*et al.* found that the addition of Na content has a strong influence on the HDS performance of Ni–Mo catalysts supported on TiO_2_ nanotubes and their reduction nature. Similar results were obtained by Sawada,^12^ in which the reduction temperatures of Rh_2_P supported on Al_2_O_3_ and SiO_2_ catalysts could be reduced by addition of Na to the corresponding catalyst. One of their explanations is that the alkali metal cation may act as a trap for dissociated hydrogen species significantly reducing hydrogen spillover and hydrogen mobility on the catalyst surface.^13^ However, to the best of our knowledge, the effect of reduction temperature on the performance of Na doped support was not discussed and role of Na is still not fully recognized. Therefore, further investigations are still needed.

In this paper, the Ni_2_P catalyst supported on the Na doped MCM-41 supports (Na-MCM-41) were successfully prepared. The aims of this research are to study the effect of reduction temperature on the structure and HDS performance of the Ni_2_P/Na-MCM-41 catalyst and propose a promising low energy consumption (a low reduction temperature) and high HDS activity preparation method of supported Ni_2_P catalysts.

## 2. Experimental

### 2.1. Preparation of support and catalysts

Siliceous MCM-41 was synthesized using tetraethyl orthosilicate (TEOS) as the silica source and cetyltrimethylammonium bromide (CTAB) as the template, following the procedure as described in the literature.^14^ The Na-MCM-41 was prepared following the method reported by our previous study.^15^ The supports obtained were named ‘M41’ for MCM-41 and ‘Na-M41’ for Na-MCM-41, respectively, where the mass fraction of Na is 0.7 wt%. The supported Ni_2_P catalysts were prepared by TPR method. In a typical synthesis technique, 2.66 g (NH_4_)_2_HPO_4_ and 2.95 g Ni(NO_3_)_2_·6H_2_O were dissolved in 20 mL of deionized water at room temperature to form a uniform solution. A quantity of 4.8 g Na-M41 was wet impregnated with the above solution for 12 h. After the water was evaporated, the resulting solid was dried at 120 °C for 12 h and calcined at 500 °C for 3 h to obtain the final oxidic precursor. It was then ground with a mortar and pestle, pelletized using a press, crushed, and then sieved to achieve a particle diameter of 16/20 mesh, finally the samples were transferred to a crucible and placed in the center of the tube furnace, and then reduced by heating from room temperature room temperature to 450 °C, 500 °C, 550 °C and 600 °C at a rate of 2.0 °C min^−1^ in a flow of H_2_ (200 mL min^−1^), and held for 2 h at each temperature.^16^ The obtained catalysts were allowed to cool naturally to room temperature in a continuous H_2_ flow and then passivated in an O_2_/N_2_ mixture (0.5 vol% O_2_) with a flow rate of 20 mL min^−1^ for 2 h. The obtained catalysts with the Ni loading of 9.73 wt%, an initial Ni/P molar ratios of 1/2 are denoted as Ni_2_P/Na-M41-*T*, where *T* is the reduction temperature of Ni_2_P/Na-M41 catalyst precursors.

### 2.2. Catalysts characterization

The reducibility of precursors was characterized by the H_2_ temperature programmed reduction (H_2_-TPR) using PC-1200 gas adsorption analyser. For the other characterizations, the reduced and passivated catalysts were immediately sealed and were characterized as soon as possible. The X-ray diffraction (XRD) patterns were obtained with a D/max-2200PC-X-ray diffractometer using Cu Kα radiation under the setting conditions of 40 kV, 30 mA, scan range from 10 to 80° at a rate of 10° min^−1^. N_2_ adsorption–desorption was carried out on a NOVA2000e instrument at 77 K. The CO uptake measurements were used to titrate the surface nickel atoms and to provide an estimation of the active sites on the catalysts. The CO uptakes were obtained by pulsing calibrated volumes of CO into a He carrier. Usually, 0.2 g of sample was loaded into a quartz reactor and pretreated in H_2_ at 400 °C for 3 h. After cooling in He, pulses of CO in a He carrier at 40 cm^3^ (NTP) min^−1^ were injected at RT through a sampling valve. CO uptake was calculated by measuring the decrease in the peak areas caused by adsorption in comparison with the area of a calibrated volume.

The analysis of the TEM images of the Ni_2_P particles can be done with the open source program ImageJ, with which the particles can be measured. The scale must be transferred and then the primary particles can be measured. The X-ray photoelectron spectroscopy (XPS) spectra were acquired using ESCALAB MKII spectrometer.

### 2.3. Catalytic activity tests

The HDS of DBT over prepared catalysts was performed in a flowing high-pressure fixed-bed reactor using a feed consisting of a decalin solution of DBT (1 wt%), WHSV = 2.5 h^−1^, and hydrogen/oil ratio of 550 (v/v). Prior to reaction, 0.8 g of the catalysts were pretreated *in situ* with flowing H_2_ (30 mL min^−1^) at test temperature for 2 h. Sampling of liquid products was started 2 h after the steady reaction conditions had been achieved. The feed and reaction product was analyzed by FID gas chromatography with a GC-14C-60 column. Turnover frequency (TOF) values of the samples containing nickel phosphide were calculated using eqn (1):^17^

1 TOF = (*F* × *X*)/(*W* × *M*)

where *F* is the molar rate of DBT fed into the reactor (mol s^−1^), *W* is the weight of catalyst (g), *X* is the conversion of DBT (%), and *M* is the mole of sites loaded which is decided by the CO uptake.

## 3. Results and discussion

### 3.1. H_2_ temperature programmed reduction

**Fig. 1:**
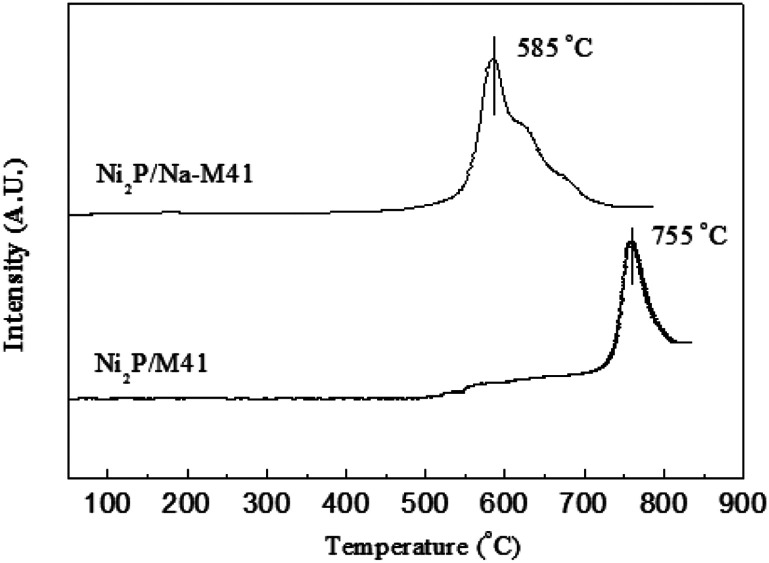
H_2_-TPR profiles of the Ni_2_P/Na-M41 catalyst precursors.

The H_2_ temperature programmed reduction (H_2_-TPR) profile of the Ni_2_P/Na-M41 catalyst precursor is shown in Fig. 1. It can be observed from Fig. 1 that the reduction of Ni_2_P/M41 precursors started at 700 °C and the hydrogen consumption peak was centered around 755 °C, which can be attributed to the reduction of highly stable P–O–P bond and the co-reductions of the nickel species in phosphate.^16^ whereas the reduction of Ni_2_P/Na-M41 precursors started at 500 °C and the hydrogen consumption peak was centered around 585 °C. This result indicates the reduction temperature of Ni_2_P/Na-M41 decreased at least 170 °C as compared to that of Ni_2_P/M41 consumption peak was centered around 585 °C. This result indicates the reduction temperature of Ni_2_P/Na-M41 decreased at least 170 °C as compared to that of Ni_2_P/M41. This reveals that the Ni_2_P phase obtained could be obtained under lower temperature by doping Na. One explanation^12^ was that (NH_4_)_2_HPO_4_ could be transformed to polyphosphate during the calcination process. Generally, a certain number of the oxygen atoms are shared between PO_4_ groups in polyphosphates, and the reduction of polyphosphates were realized by breaking of P–O–P linkages, which is more difficult as compared with the P–O–H linkages in phosphate. Van Wazer and co-workers^17^ studied the effect of Na cation on the distribution of phosphate structural units on the basis of statistics. They found that Na cation acts as a polymerization inhibitor, which decrease the length of chain phosphates. It was also reported^18^ that the metal Na is partially covalently bonded in the complexes or at least are held at specific sites, which further demonstrated that the formation of polyphosphate with highly stable P–O–P bond could be prevented by the addition of Na. Recently, Sawada *et al.*^12^ found that the precursor of phosphide catalyst required reduction temperature can be decreased by Na addition. As our results showed similar results to the reported literatures, we can also attributed the decreased temperature of hydrogen consumption peak to polymerization inhibitor Na, which makes the phosphide catalyst be easily formed at a lower reduction temperature.

### 3.2. The X-ray diffraction (XRD) patterns analysis

**Fig. 2:**
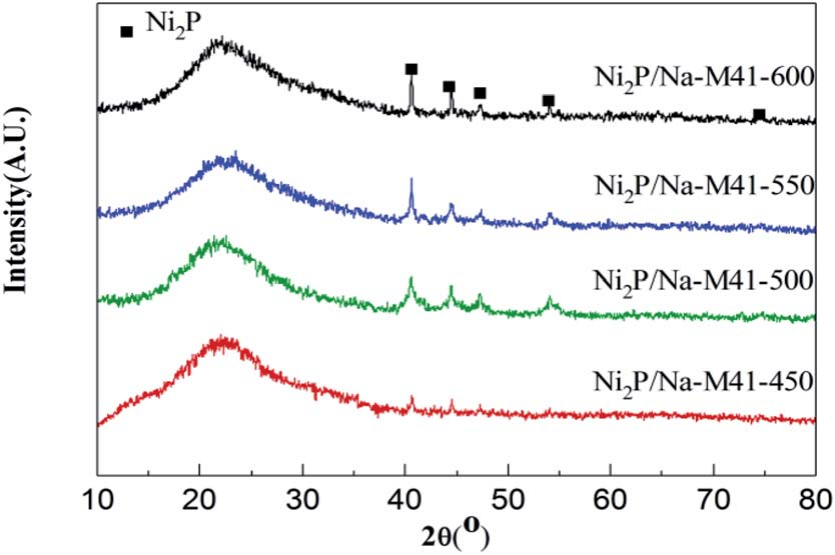
XRD patterns of the Ni_2_P/Na-M41-*T*.

The X-ray diffraction (XRD) patterns of Ni_2_P/Na-M41-*T* catalysts are shown in Fig. 2. As can be seen, all samples exhibit a broad feature owing to the amorphous nature of mesoporous M41 at 2*θ* ≈ 22°. For the Ni_2_P/Na-M41-*T* samples, the strong diffraction peaks at 2*θ* = 40.6°, 44.5°, 47.1° and 54.1° (PDF: 03-0953) can be assigned to Ni_2_P, and no additional phase related to Ni and P is observed. This indicates that the active phase formed is mainly Ni_2_P for these samples. It is worth noting that some obvious diffraction peaks of Ni_2_P phase were started to generate at the reduction temperature of 450 °C, indicating that the addition of Na can decrease the reduction temperature of Ni_2_P, which is about 200 °C lower than that of the conventional temperature-programmed reduction (TPR) method (650–1000 °C).^19^ This result is in accordance with the H_2_-TPR analysis (Fig. 2). With increasing the temperature from 450 °C to 600 °C, the Ni_2_P phase peaks become more intense and sharpen, showing that more Ni_2_P particles were formed and the crystal sizes of Ni_2_P phase were increased with increasing the reduction temperature formation of the active Rh_2_P phase. The crystallite sizes (*D*_c_) of Ni_2_P phase were calculated from Scherrer's equation^20^ and listed in column 5 of Table 1. *D*_c_ increased with increasing the reduction temperature from 450 to 600 °C, and it will be demonstrated again in the next analysis.

**Table 1:** The properties and HDS catalytic performance of the support and catalysts

Sample	*S*_BET_ (m^2^ g^−1^)	*V*_p_ (cm^3^ g^−1^)	*d* (nm)	*D*_c_ (nm)	CO uptake (μmol g^−1^)	Conversion (%) 280 °C	Selectivity (%)		TOF (10^−3^·s^−1^)
							CHB	BP	
M41	911	0.962	4.2	—	—	—	—	—	—
Na-M41	858	0.874	4.1	—	—	—	—	—	—
Ni_2_P/Na-M41-450	315	0.349	4.5	8.8	27	40.2	11.1	88.9	3.2
Ni_2_P/Na-M41-500	287	0.316	4.4	9.6	40	59.1	12.2	87.8	3.4
Ni_2_P/Na-M41-550	135	0.160	7.7	19.7	48	65.9	12.4	87.6	3.8
Ni_2_P/Na-M41-600	122	0.144	9.7	20.9	51	69.3	15.4	84.6	3.9

### 3.3. Transmission electron microscope examinations

**Fig. 3:**
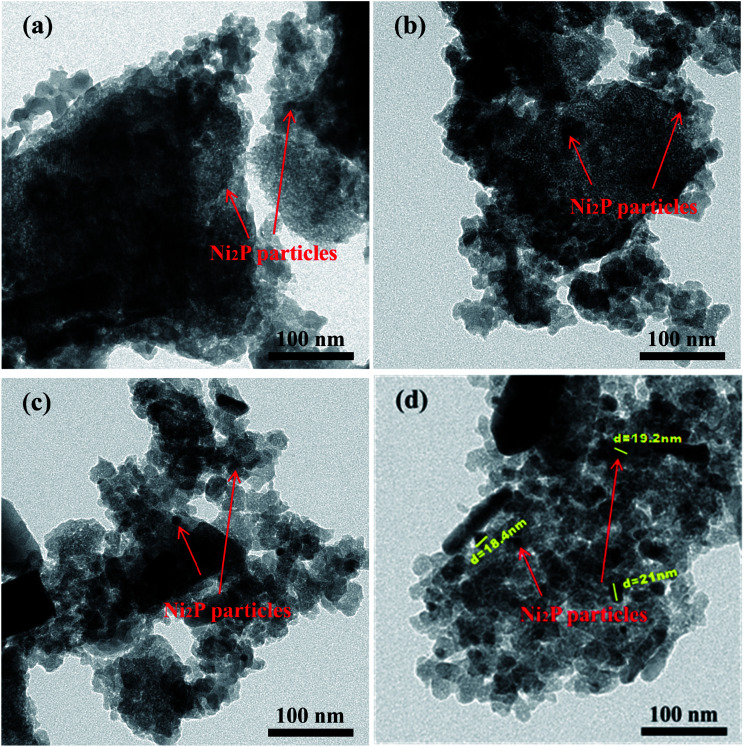
TEM spectra of (a) Ni_2_P/Na-M41-450, (b) Ni_2_P/Na-M41-500, (c) Ni_2_P/Na-M41-550, and (d) Ni_2_P/Na-M41-600.

**Fig. 4:**
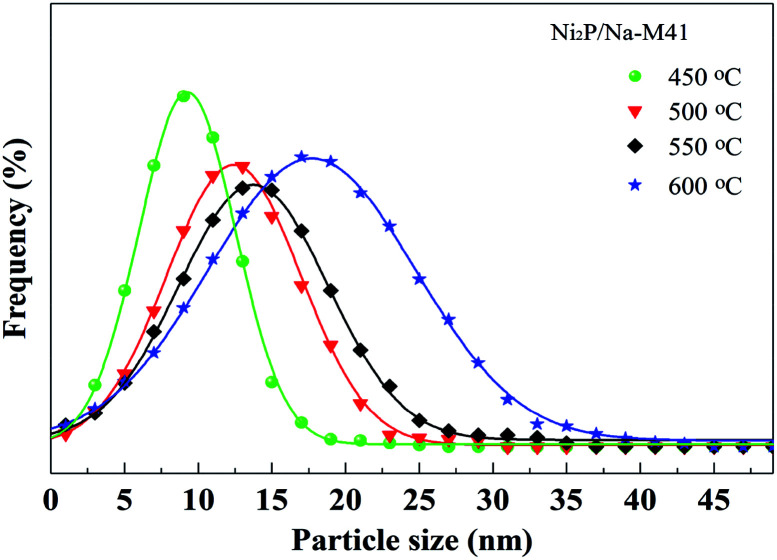
Particle size distribution of Ni_2_P/Na-M41-*T* catalysts.

Transmission electron microscope (TEM) images and the particle size distribution of the Ni_2_P/Na-M41-*T* catalysts are shown in Fig. 3 and 4. In order to measure the size of the catalyst more accurately, the size of about 400 Ni_2_P particles in TEM image were measured by ImageJ software. The average Ni_2_P particle size in the Ni_2_P/Na-M41-450 and Ni_2_P/Na-M41-600 catalyst was 8 nm and 16.2 nm respectively. These results reveal that an increase in reduction temperature leads to sintering of the catalyst and the formation of larger particles. Moreover, unlike the typical stacked morphologies of Mo and W sulfides, Ni_2_P are not layered and form spherical particles that can be well dispersed on supports.^21^ With increasing the reduction temperature, the average Ni_2_P particle size increased, which was consistent with observation from XRD analysis.

### 3.4. Textural properties

**Fig. 5:**
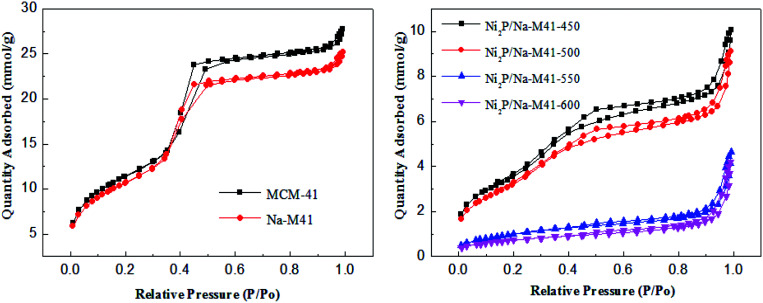
The nitrogen adsorption/desorption isotherms for M41, Na-M41, and Ni_2_P/Na-M41-*T* catalysts.

All the results of the textural properties of the Na-M41 support and the various catalysts prepared using different reduction temperatures are listed in Table 1. In general, the BET surface areas of the Na doped support M41-41 (858 m^2^ g^−1^) were lower than that of bare support (911 m^2^ g^−1^), a similar trend was observed with the pore volumes and the average pore size of the support, which can be attributed to the incorporation of Na onto the support material. Moreover, there is a noticeable difference in the magnitude of the specific surface area of the catalysts depending on the applied reduction temperature. The surface area of the catalysts decreased from 315 to 122 m^2^ g^−1^ when the reduction temperature increased from 450 to 600 °C. However, Ni_2_P/Na-M41-600 has the largest average pore diameter of 9.7 nm compared with the others, this is possibly because the higher reduction temperature may destroy the pore of catalysts. As shown in Fig. 5, the shapes of the adsorption–desorption isotherms of Ni_2_P/Na-M41-*T* are type of IV isotherm and a standard H4 type hysteresis loop according to the IUPAC classification, which showed that some mesopores are presented for all samples.

### 3.5. CO uptake analysis

The CO uptakes was used to estimate the surface density of exposed Ni sites on the catalysts.^22^ The CO uptakes at room temperature of the Ni_2_P/Na-M41-*T* catalysts are listed in column 6 of Table 1. As common sense, CO molecules may also be adsorbed on P sites, the amount of CO molecules may be very small and can be ignored.^23^ As shown in Table 1, the CO adsorption of the Ni_2_P/Na-M41-450 catalyst was 27 μmol g^−1^. Upon increasing the reduction temperature of the catalysts, the CO uptakes of the Ni_2_P/Na-M41-*T* samples were significantly increased, which showed that increasing the reduction temperature can lead to an increase in the amount of exposed nickel atoms on the surface. This may be ascribed to the formation of more Ni_2_P particles (XRD analysis) and suppression of the P enrichment on the surface (XPS analysis) with increasing the reduction temperature. This will be discussed further with the XPS analysis.

### 3.6. The X-ray photoelectron spectroscopy spectra

**Fig. 6:**
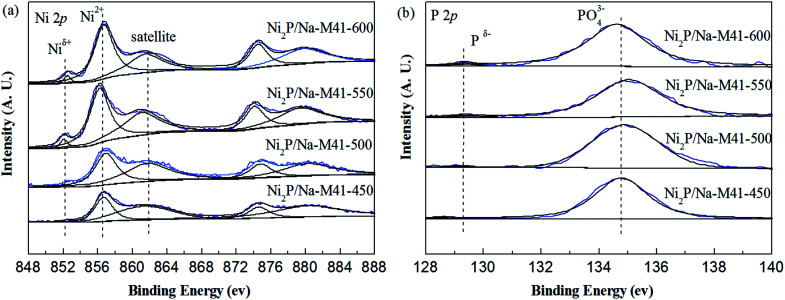
XPS spectra of the Ni_2_P/Na-M41-*T* catalysts.

In order to gain further insight into the surface composition of the samples and the valence states of the active components, the XPS spectra technique of samples was performed. The XPS spectra of the Ni_2_P/Na-M41-*T* samples in the Ni (2p) and P (2p) regions are shown in Fig. 6, and the binding energies for all samples are listed in Table 2. As shown in Fig. 6(a), all spectra were decomposed, taking into account the spin-orbital splitting of the Ni 2p_3/2_ and Ni 2p_1/2_ lines (about 17 eV) and the presence of satellite peaks at about 5 eV higher than the binding energy of the parent signal.^24^ As is well know that Ni 2p_3/2_ core-level spectrum consists of three components. The bands centered at 852.1–852.5 eV can be ascribed to Ni^*δ*+^ in the Ni_2_P phase, and the second at 856.2–856.8 eV corresponds to the Ni^2+^ species interacting with phosphate species as a consequence of superficial passivation, accompany with the broad satellite at approximately 5.0 eV higher than that of the Ni^2+^ species and this shake-up peak is assigned to divalent species^25^ Meanwhile, other broad peaks on the high binding energy side can be ascribed to the Ni 2p_1/2_ signal from oxidized Ni species.^26^ P 2p binding energy involves the peaks at 128.9–129.3 eV can be assigned to P^*δ*−^ species on the metal phosphides^24^ and the peak at 134.6–135.0 eV due to phosphate (P^5+^) arising from superficial oxidation of nickel phosphide particles.^27^ As can be seen from Fig. 6(a) and Table 2, the XPS spectra for the as prepared Ni_2_P/Na-M41-450 exhibited Ni 2p peaks at 852.6 and 856.7 eV, which can be attributed to the Ni^*δ*+^ band forming Ni_2_P phase and Ni^2+^ species, respectively. No obvious change can be seen for the binding energy of Ni species among the Ni_2_P/Na-M41-*T*. In addition, the intensity of Ni^*δ*+^ species bands of the Ni_2_P/Na-M41-*T* catalyst become more intense with increasing the reduction temperature. Fig. 6(b) shows the peaks of Ni_2_P/Na-M41-450 centered at 134.8 eV for PO_4_^3−^ was observed, together with a low intensity peak at 128.9 eV that can be attributed to the P^*δ*−^ band forming Ni_2_P. The intensity of P^*δ*+^ species bands over the Ni_2_P/Na-M41-*T* catalyst became more intense with increasing the reduction temperature, which showed the similar tendency with the Ni^*δ*+^ species in Ni 2p. Therefore, it can be concluded that increasing the reduction temperature of the precursors can promote the formation of more Ni_2_P particles. The binding energy of P^*δ*−^ is increased with increasing the reduction temperature. This special effect is beneficial to the reduction PO_3_^4−^ into Ni_2_P.

**Table 2:** Spectral parameters obtained by XPS analysis for the catalysts

Sample	Binding energy (eV)					Superficial atomic ratio			
	Ni 2p_3/2_			P 2p_3/2_					
	Ni^2+^	Satellite	Ni^*δ*+^	P^5+^	P^*δ*−^	P/Ni	Na (%)	Ni (%)	Na/Ni
Ni_2_P/Na-M41-450	856.7	861.8	852.4	134.8	128.9	1.99	0.89	4.72	0.19
Ni_2_P/Na-M41-500	856.8	861.7	852.5	134.9	129.1	1.96	0.73	3.58	0.21
Ni_2_P/Na-M41-550	856.2	861.0	852.1	135.0	129.3	1.34	0.5	2.02	0.24
Ni_2_P/Na-M41-600	856.7	861.4	852.4	134.6	129.3	1.33	0.49	1.87	0.26

### 3.7. Hydrodesulfurization activity

**Fig. 7:**
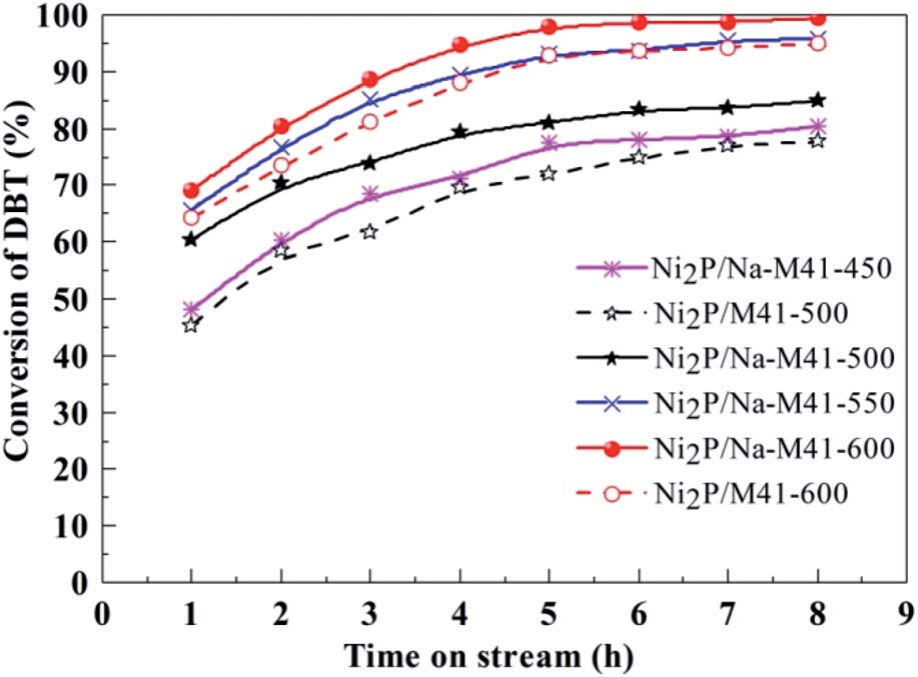
The HDS activity of the Ni_2_P/Na-M41-*T* catalysts. Temperature, 340 °C; pressure, 3.0 MPa; H_2_/oil ratio, 550 (v/v); WHSV, 2.5 h^−1^.

Fig. 7 depicts the variation of DBT conversion with time on stream during DBT HDS catalyzed by Ni_2_P/Na-M41-*T* catalysts. As can be seen from Fig. 7, the HDS activities for all samples gradually increased with time on stream initially, and 99.2% DBT conversion was observed for Ni_2_P/Na-M41-600 after 8 h, during which more active intermediate phase gradually formed, and then tended to stable, showing the active intermediate phase had completely formed. The active intermediate phase is regarded as a superficial phosphosulfide with a stoichiometry represented by NiP_*x*_S_*y*_, which is more active than Ni_2_P phase.^27^

As seen from Fig. 7, the DBT conversion of Ni_2_P/Na-M41-500 catalyst reduced at 500 °C was 84.7% after 8 h, which was 7.2% higher as compared to that of Ni_2_P/M41-500 prepared at the same reduction temperature. This reveals that the benefit of the addition of Na is that a higher HDS activity can be achieved at lower reduction temperature. Furthermore, it is worth noting that the DBT conversions of Ni_2_P/Na-M41-*T* catalyst increased upon increasing the reduction temperature and nearly 100% DBT conversion (99.2%) was observed for Ni_2_P/Na-M41-600. While for the Ni_2_P/M41-600 the DBT conversion is only 95.0%, which is low. The CO uptakes can be used to evaluate the TOF of the catalyst (Table 1). The HDS TOF of Ni_2_P/Na-M41-*T* increased with increasing reduction temperature. And the TOF of Ni_2_P/Na-M41-600 reached 3.69 × 10^−3^ s^−1^, indicating more effective Ni_2_P phase was formed at higher reduction temperature. Therefore, the boosted HDS activity observed in the Ni_2_P/Na-M41-*T* catalysts could be attributed to the highly dispersed active Ni_2_P phase, as well as lower coverage of phosphorus on the surfaces of these catalysts (Table 1, XPS analysis).

To investigate the effect of reduction temperature on the HDS catalytic selectivities, the selectivities to BP and CHB over Ni_2_P/Na-M41-*T* catalysts are presented in Table 1. For all the samples, BP is formed in greater proportions, indicating that DBT primarily removed by the DDS pathway over all the catalysts. The Ni_2_P/Na-M41-450 has a BP selectivity of 88.9%, and the BP selectivities of catalysts show a slightly decreased with increasing the reduction temperature, which indicates the lower reduction temperature favored the DDS route.

## 4. Conclusions

In this research the Ni_2_P/Na-M41-*T* catalysts with Na loading of 0.7 wt%, Ni loading of 9.73 wt% and an initial Ni/P molar ratios of 1/2 were successfully prepared. The effect of reduction temperature (450–600 °C) on the structure and HDS performance of Ni_2_P/MCM-41 catalyst was studied. TPR result indicates the reduction temperature of Ni_2_P/Na-M41 is decreased at least 170 °C as compared to that of Ni_2_P/M41 owing to the incorporation of Na, which would save energy consumption during preparation of catalyst. The decreased reduction temperature can be attributed to the formation of polyphosphate with highly stable P–O–P bond could be prevented by the addition of Na. The XRD analysis exhibited that the Ni_2_P/Na-M41 catalyst can be obtained at reduction temperature of 450 °C, which is about 200 °C lower than that of the conventional TPR method (650–1000 °C). Increasing the reduction temperature lead to an increase in the Ni_2_P particle size and decrease the enrichment of P on the surface, and therefore give rise to more exposed nickel sites.

The DBT conversions of Ni_2_P/Na-M41-*T* catalyst increased upon increasing the reduction temperature and nearly 100% DBT conversion (99.2%) was observed for Ni_2_P/Na-M41-600, which is higher than that of the Ni_2_P/M41-600 (95.0%) prepared by conventional TPR method. This can be attributed to the highly dispersed active Ni_2_P phase, as well as lower coverage of phosphorus on the surfaces of these catalysts. The DBT primarily removed by the DDS pathway over all the catalysts and the lower reduction temperature favored the DDS route.

## Conflicts of interest

There are no conflicts to declare.

## Supplementary Material

## References

[cit1] Umar C. A., Khalid R. A., Sagir A. (2019). J. Cleaner Prod..

[cit2] Jang J. G., Lee Y. K., Wu P. Y. (2019). Appl. Catal., B.

[cit3] Zhao H. Y., Oyama S. T., Freund H. J., Włodarczyk R., Sierka M. (2015). Appl. Catal..

[cit4] Liang J. L., Wu M. M. (2018). J. Catal..

[cit5] Oyama S. T., Zhao H., Freund H. J., Asakura K., Włodarczyk R. (2012). J. Catal..

[cit6] Tian S., Li X., Wang A., Prins R. (2016). Angew. Chem., Int. Ed..

[cit7] Jiménez-Gómez C. P., Cecilia J. A., Moreno-Tost R. (2017). ChemCatChem.

[cit8] Hsu P. J., Lin Y. C. (2017). J. Taiwan Inst. Chem. Eng..

[cit9] Yu Z. Q., Wang A. J., Liu S. (2019). Catal. Today.

[cit10] Mendeza F. J. (2017). Appl. Catal., B.

[cit11] Rodrigo A., Ortega-Domínguez R. A. (2015). J. Catal..

[cit12] Sawada A., Kanda Y. (2014). Catal. Commun..

[cit13] Dong C., Li X. (2017). Catal. Today.

[cit14] Meléndez-Ortiz H. I., García-Cerda L. A., Olivares-Maldonado Y., Castruita G., Mercado-Silva J. A. (2012). Ceram. Int..

[cit15] Song H., Dai M., Song H. L., Wan X., Xu X. W., Jin Z. S. (2014). J. Mol. Catal. A: Chem..

[cit16] Song H., Zhang F. Y., Jiang N., Chen M. S., Li F., Yan Z. J. (2018). Res. Chem. Intermed..

[cit17] Parks J. R., Van Wazer J. R. (1957). J. Am. Chem. Soc..

[cit18] John R., Van Wazer J. R. (1958). Chem. Rev..

[cit19] Song H., Dai M., Song H. L., Wan X., Xu X. (2013). Appl. Catal., A.

[cit20] Oyama S. T., Wang X., Lee Y. K., Chun W. J. (2004). J. Catal..

[cit21] Song H., Dai M., Song H. L., Wan X., Xu X. (2013). Appl. Catal., A.

[cit22] Goncalvesa V. O. O., Souza P. M. D. (2017). Appl. Catal., B.

[cit23] Guan Q. X., Wan F. F., Han F., Liu Z. H., Li W. (2016). Catal. Today.

[cit24] Yun G. X., Guan Q. X., Li W. (2018). J. Catal..

[cit25] Song H., Gong J., Song H. L., Li F. (2015). Appl. Catal., A.

[cit26] Song L., Zhang S., Wei Q. (2011). Catal. Commun..

[cit27] Song H., Dai M., Guo Y. T. (2012). Fuel Process. Technol..

